# A Novel Adeno-Associated Virus–Based Genetic Vaccine Encoding the Hepatitis C Virus NS3/4 Protein Exhibits Immunogenic Properties in Mice Superior to Those of an NS3-Protein-Based Vaccine

**DOI:** 10.1371/journal.pone.0142349

**Published:** 2015-11-10

**Authors:** Fengqin Zhu, Tian Chen, Yeqiong Zhang, Haixia Sun, Hong Cao, Jianxi Lu, Linshan Zhao, Gang Li

**Affiliations:** Department of Infectious Diseases, The Third Affiliated Hospital, Sun Yat-Sen University, Guangzhou, People’s Republic of China; University of North Carolina at Chapel Hill, UNITED STATES

## Abstract

More than 170 million individuals worldwide are infected with hepatitis C virus (HCV), and up to an estimated 30% of chronically infected individuals will go on to develop progressive liver disease. Despite the recent advances in antiviral treatment of HCV infection, it remains a major public health problem. Thus, development of an effective vaccine is urgently required. In this study, we constructed novel adeno-associated virus (AAV) vectors expressing the full-length NS3 or NS3/4 protein of HCV genotype 1b. The expression of the NS3 or NS3/4 protein in HepG2 cells was confirmed by western blotting. C57BL/6 mice were intramuscularly immunised with a single injection of AAV vectors, and the resultant immune response was investigated. The AAV2/rh32.33.NS3/4 vaccine induced stronger humoral and cellular responses than did the AAV2/rh32.33.NS3 vaccine. Our results demonstrate that AAV-based vaccines exhibit considerable potential for the development of an effective anti-HCV vaccine.

## Introduction

Hepatitis C virus (HCV) infection is a major public health problem affecting more than 170 million people worldwide and is a leading cause of cirrhosis, hepatocellular carcinoma, and liver failure [1]. Treatment for HCV has progressed rapidly, especially for genotype 1. Previously, the standard treatment for HCV genotype 1 infection is peginterferon plus ribavirin, with a SVR rate of less than 50%. Then from 2011, combination of the protease inhibitor with peginterferon and ribavirin increased the sustained virological response (SVR) rates to 70% for untreated HCV genotype 1 infection [2, 3]. Since 2014, the interferon-free regimen of ledipasvir/sofosbuvir (Harvoni,Gilead Sciences) resulted in more than 95% SVR rates in patients with HCV 1b infection [4, 5]. However, high cost of these treatments highly limited their access in developing countries, where the disease burden is greatest. Nowadays, no effective vaccine has been demonstrated to date despite identification of several vaccine candidates in preclinical and clinical trials [6–10]. Therefore, development of an effective, safe, and affordable anti-HCV vaccine is a matter of great urgency.

HCV is a positive-strand RNA virus of the *Flaviviridae* family, which exists as seven major genotypes and several subtypes [11]. Genotype 1b HCV is the most prevalent form worldwide, particularly in Europe and East Asia. When HCV enters the cytoplasm, the viral RNA genome is translated into a polyprotein that undergoes proteolytic cleavage by cellular and viral proteases into three structural viral proteins (core, E1, and E2), a small membrane polypeptide (p7), and six non-structural proteins (NS2, NS3, NS4A, NS4B, NS5A, and NS5B) [12–14]. The NS3/4 protein complex of HCV has important protease and helicase activities and participates in the replication module with NS5A and NS5B. A set of CD4^+^ T-cell epitopes has been identified within the NS3/4 region; these epitopes may be optimal candidates for use in immunotherapy for HCV infection [15]. The NS3 protein has important protease and RNA helicase activities. Multiple CTL epitopes have been identified in the NS3 region, the cellular immune response against which determines the viral persistence outcome [16]. Several studies have shown that NS3-specific T-cell responses correlate with resolution of acute HCV infection [17–19]. Therefore, NS3 may be an ideal candidate for a novel vaccine [20].

Viral vectors expressing foreign antigens are widely used to induce T-cell immunity against pathogens [21–23]. The use of adeno-associated virus (AAV) vaccines has recently become an attractive approach because of the capacity of these agents to persist for prolonged periods in the transduced tissues [24, 25]. AAV is a single-stranded DNA virus belonging to the *Parvoviridae* family. Because of its non-pathogenicity, ability to transduce both dividing and non-dividing cells, and relatively low immunogenicity, AAV has been explored as a vector for gene therapy. It has been identified as safe in human clinical trials and effective in the treatment of rare inherited diseases [26]. More than 120 serotypes and variants from human and nonhuman primates have been identified to date. Many novel capsids isolated from primates show high transduction efficiency and low seroprevalence [27]. For example, AAVrh32.33, a novel vector developed from rhesus macaque isolates, has lower seroprevalence in human populations than do AAV2 and AAV8 and has been evaluated as a genetic platform for an HIV-1 vaccine. Robust CD8^+^ T-cell responses to the HIV gag and gp140 proteins were observed in mouse and macaque models, respectively [24]. Our laboratory has developed genetic vaccines based on AAV vectors expressing truncated dengue virus envelope proteins, which induced a long-lasting humoral response in mice [28]. In the present study, we constructed AAV2/rh32.33 vectors expressing the NS3 or NS3/4 protein of HCV genotype 1b and evaluated their immunogenicity using a mouse experimental model.

## Materials and Methods

### Plasmid construction

Total RNA of HCV genotype 1b (GenBank accession number AB049099) was obtained from the serum of a patient diagnosed with HCV (viral load = 2.57E+08 IU) in the Third Affiliated Hospital of Sun Yat-Sen University (Guangzhou, China) using the Mini BEST Viral RNA/DNA Extraction Kit ver. 4.0 (TaKaRa) according to the manufacturer’s protocol. cDNA was synthesised using the PrimeScript II First-Strand cDNA Synthesis Kit (TaKaRa). To amplify the NS3 gene, PCR using Pyrobest DNA polymerase (Takara) and specific primers was carried out, and *Not*I and *EcoR*I sites were used to clone the amplicons into the AAV cis-plasmid (a kind gift from J. Wilson of University of Pennsylvania).Sequences were as follows: forward, 5′GTGGAATTCATGGCGCCTATCACGGCCTAT3′; and reverse, 5′CCACGCGGCCGCGACGACCTACAG3′. The NS3/4 gene cDNA was amplified by nested PCR using PrimeSTAR^®^ HS DNA Polymerase with GC Buffer (TaKaRa) and specific primers (Table 1). The segment was then cloned into the AAV cis-plasmid using an In-Fusion HD Cloning Kit (Clontech) and two specific primers (Table 1). The identification of AAV recombinants was confirmed by restriction enzyme digestion and sequencing. The clones were named pAAV.CMV.HCV.NS3 and pAAV.CMV.HCV.NS3/4. The study was approved by the Ethics Committee of the third affiliated hospital of Sun Yat-sen University (Guangzhou, China) and written informed consent was obtained.

**Table 1:** Primers for the amplification of HCVNS3/4 gene.

Primer name	Sequences
s3366	5'GAGATACTYYTRGGRCCRGCYGATR3'
a6300	5'CCGWGCATATCCADTCCCARAYRTC3'
s3420	5'ATGGCSCCCATCACRGCYTATYCCC3'
a6257	5'TCARCAYGGYGTGGARCAGTCCTCATTR3'
F3420	5’GGTGTCCAGGCGGCCGCATG3’
F6257	5’CAGTCGAGGCAGATCTTCAACATGGTGTG3’

s3366 and a6300 were primers for the first round PCR; s3420 and a6257 were primers for the second round PCR.F3420 and F6257 were primers for In-Fusion Cloning.

### AAV preparation, purification, and quantification

AAV particles were produced by transfecting a three-plasmid vector system into HEK-293 cells (a kind gift from J. Wilson of University of Pennsylvania), as described previously [29, 30]. The plasmid system comprises one transfer plasmid (pAAV.CMV.eGFP, pAAV.CMV.NS3, or pAAV.CMV.NS3/4) and two packaging plasmids (pAAV2/rh32.33 and pAd.F6). All plasmids were extracted using a Plasmid Maxi Kit (Qiagen) as instructed by the manufacturer. Briefly, 2 h before transfection, each 15-cm-diameter plate containing 293 cells (70%–80% confluent) was treated with 20 ml of fresh Dulbecco’s modified Eagle’s medium (DMEM; Gibco) containing 10% foetal bovine serum (FBS; Hyclone) without antibiotics. Equimolar plasmids were dissolved in 650 μl of 2.5 M CaCl_2_ and 5.9 ml of MilliQ water, then rapidly mixed with 12.5 ml of 2 × 4-(2-hydroxyethyl)-1-piperazineethanesulfonic acid (HEPES)-buffered saline (HBS; pH 7.05). A 2.5-ml aliquot of the transfection solution was gently added to each dish. The plates were swirled slowly to mix the contents, then returned to the incubator. Sixteen hours after transfection, the medium was replaced with fresh DMEM (Gibco) containing 10% FBS and 100 mg/ml streptomycin and penicillin (Gibco). Seventy-two hours later, cells were collected when green eGFP signals had become distinct-shaped foci as visualised using fluorescence microscopy. The supernatant was discarded and the pellet resuspended in 10 ml of 150 mM NaCl, 20 mM Tris (pH 8.0) per tissue culture plate. Benzonase nuclease (Sigma-Aldrich) was then added to a final concentration of 50 units/ml, and the plates were centrifuged at 3000 × *g* for 30 min at 4°C to remove cellular debris. The cells were then lysed by means of three consecutive freeze–thaw cycles (–80°C and 37°C) to release AAV. AAV was purified by three rounds of caesium chloride gradient centrifugation, and genome titres (genome copies [GC] per ml) were determined by real-time PCR using Premix Ex Taq^™^ (TaKaRa).

### Infectivity test and analysis of NS3 and NS3/4 expression

HepG2 cells were used to evaluate AAV infectivity. Cells were cultured in six-well plates with DMEM supplemented with 10% FBS and infected with rAAV2/rh32.33.NS3/4, rAAV2/rh32.33.NS3, or rAAV2/rh32.33.eGFP (negative control) for analysis of gene expression. The final infection concentration of each virus was 1 × 10^10^ GC/ml. Seventy-two hours later, after confirmation of eGFP expression by fluorescence microscopy, the infected cells were harvested and lysed for western blotting analysis. Samples of the cell lysates were separated in 10% and 14% SDS-PAGE gels and then transferred onto polyvinylidene fluoride membranes (Millipore). Nonspecific binding sites were blocked by incubating membranes in 5% non-fat milk. Blots were incubated with an anti-HCV NS3 mouse monoclonal antibody (Virostat 1878), anti-HCV NS4A mouse monoclonal antibody (Virostat 1866), and anti-HCV NS4B mouse monoclonal antibody (Abcam,ab24283) overnight at 4°C. The membranes were then washed three times in TBST and incubated with the horseradish peroxidase (HRP)-conjugated secondary antibody (Abcam) for 1 h at room temperature. The membranes were washed as above, and the proteins were visualised using Dura Super Signal Substrate (Pierce, USA).

### Immunisation of mice

C57BL/6 mice (female, 4–6 weeks old) were purchased from SLAC Laboratory Animal Company (Shanghai, China). They were randomly divided into 4 groups of 12 mice each. Vaccination schedules were as follows: group 1, immunisation with the recombinant AAV2/rh32.33.NS3/4 vaccine (5 × 10^11^ GC per mouse); group 2, immunisation with the recombinant AAV2/rh3233.NS3 vaccine (5 × 10^11^ GC per mouse); group 3, immunisation with the recombinant AAV2/rh3233.eGFP vaccine (5 × 10^11^ GC per mouse); and group 4 (control), injection of PBS (100 μl per mouse). Each mouse was vaccinated intramuscularly at the tibialis anterior muscle of the hind leg. Blood samples were collected from the retro-orbital sinus of six mice selected randomly at weeks 0, 4, 8, and 12 after immunisation. In addition, at week 4 post-immunisation, splenocytes of a further six mice were isolated according to standard techniques under deep anaesthesia. The animal protocols used in this work were evaluated and approved by the Animal Use and Ethics Committee (CEUA) of the Third Affiliated Hospital, Sun Yat-Sen University (Protocol 2012–0083).

### ELISA for NS3-specific antibody reactivity

To measure antibody reactivity against the immunised antigens, 96-well Nunc ELISA plates were coated with GST-NS3 peptide and incubated overnight at 4°C. The plates were washed and blocked with 1% BSA for 2 h at room temperature and then washed three times with PBST. A fixed volume (100 μl/well) of 1:100 diluted serum samples was tested. The plates were incubated for 2 h at 37°C and washed five times, and then 1:1,000 diluted anti-mouse IgG HRP-conjugated antibody (Sigma) was added followed by incubation for 1 h at 37°C. After washing six times with PBST, 3,3′,5,5′-tetramethylbenzidine (TMB; Invitrogen) was added to each well, followed by incubation for 15 min at room temperature; the plates were then read (ELX800, Bioteck) at a 450-nm wavelength.

### Determination of cytokine mRNA levels in splenocytes by real-time PCR

Levels of cytokine (IFN-γ, IL-2, and IL-4) mRNA in splenocytes were measured by real-time RT-PCR. Spleens were isolated from mice at 4 weeks post-immunisation. A single splenocyte suspension was prepared by macerating the spleens through 70-μm cell strainers (Falcon) and maintained in Roswell Park Memorial Institute 1640 medium containing 10% FBS, penicillin (100 U/ml), streptomycin (100 U/ml), and 2 mM L-glutamine. Splenocytes were seeded in 96-well plates at 4 × 10^5^ cells per well in 200-μl complete medium and stimulated with 4 μg/well GST-NS3 peptide, or PBS as a control. Cultures were incubated at 37°C in 5% CO_2_ for 72 h. The cells were then harvested by centrifugation at 3000 × *g* for 5 min. Total RNA was extracted from the stimulated and control splenocytes using TRIzol reagent (Invitrogen). Real-time RT-PCR was performed using a TaKaRa RNA PCR Kit (AMV) ver. 3.0 and SYBR Premix Ex Taq (Takara). Primer sequences are shown in Table 2.

**Table 2:** Primer sequences for IFN-γ, IL-2, IL-4 and GAPDH.

Cytokine	Primer sequences
IFN-γ	Forward:5’CCTGCGGCCTAGCTCGA3’
	Reverse:5’CAGCCAGAAACAGCCATGAG3’
IL-2	Forward:5’GCACCCACTTCAAGCTCCA3’
	Reverse:5’AAATTTGAAGGTGAGCATCCTG3’
IL-4	Forward:5’GGTCTCAACCCCCAGCTAGT3’
	Reverse:5’GCCGATGATCTCTCAAGTGAT3’
GAPDH	Forward:5’CATCGCCTTCCGTGTTCCTA3’
	Reverse:5’GCGGCACGTCAGATCCA3’

### Determination of splenocyte IFN-γ and IL-4 levels by ELISA

Splenocytes were cultured and stimulated as above. Seventy-two hours later, the supernatants were collected for ELISA. Production of IFN-γ and IL-4 by splenocytes stimulated with the GST- NS3 was measured using IFN-γ Quantikine Mouse ELISA and IL-4 Quantikine Mouse ELISA kits (R&D Systems, Minneapolis, MN) according to the manufacturer’s instructions. Optical densities were determined at a wavelength of 450 nm.

### Determination of T lymphocyte proliferation by MTT assay

We determined the T lymphocyte proliferation by MTT assay (Sigma). Briefly, Splenocytes (5×10^4^/well) from vaccinated mice were pulsed with GST-NS3 peptide for 72h and exposed to MTT (5 mg/ml) for 4 h [31]. After being incubated at 37°C in 5% CO2 for 10 min, then removed the medium and added 150ul dimethyl sulfoxide (DMSO, Sigma) to each well of the plate. The optical density (OD) value was determined at a wavelength of 490 nm after being incubated at 37°C for 10 min. The stimulation index (SI) was determined as: SI = OD of stimulated culture/OD of non-stimulated culture, and SI > 2 was thought to be significant [32].

### CTL Assay for NS3-specific CTL activity

Specific CTL activity was measured using the CytoTox 96 ^®^ Non-Radioactive Cytotoxicity Assay kit (Promega). P815 mouse lymphoblast-like mastocytoma cells were transfected with pAAV.CMV.NS3 plasmid using Lipofectamine^®^ 2000 (Ivitrogen). Expression of the NS3 protein was confirmed by western blot and a stable cell line expressing NS3 was selected using G418 (Gibco). The NS3-expressing P815 cells were treated with mitomycin C (25 μg/ml, Sigma-Aldrich) for 30 min and used as target cells, P815-NS3 cells. Splenocytes were prepared as above. And then they were cultured with P815-NS3 cells and GST-NS3 peptide for 5 days to engender effector cells. The optimal number of the target P815-NS3 cell was determined as 1x104 cells / 100ul. The effectors and targets were mixed in 96-well plates for 4 h at 37℃ in 5% CO2, with different E:T ratios of 50:1, 25:1, 12.5:1, and 6.25:1. The assay plate included controls as follows: effector cell spontaneous lactate dehydrogenase (LDH) release, target cell spontaneous LDH release, target cell maximum LDH release, culture medium background, and volume correction control. Released LDH was measured as instructed by the manufacturer. The percent cytotoxicity was computed by the following formula: % cytoxicity = (experimental–effector–target spontaneous)/(target maximum–target spontaneous) ×100.

### Statistical analysis

Statistical analysis was conducted and graphs produced using the Prism ver.5 software (GraphPad Software). An unpaired, two-tailed *t* test was used to determine the significance of differences.

## Results

### Construction and characterisation of recombinant AAV vectors encoding the HCV NS3 or NS3/4 protein

**Fig 1:**
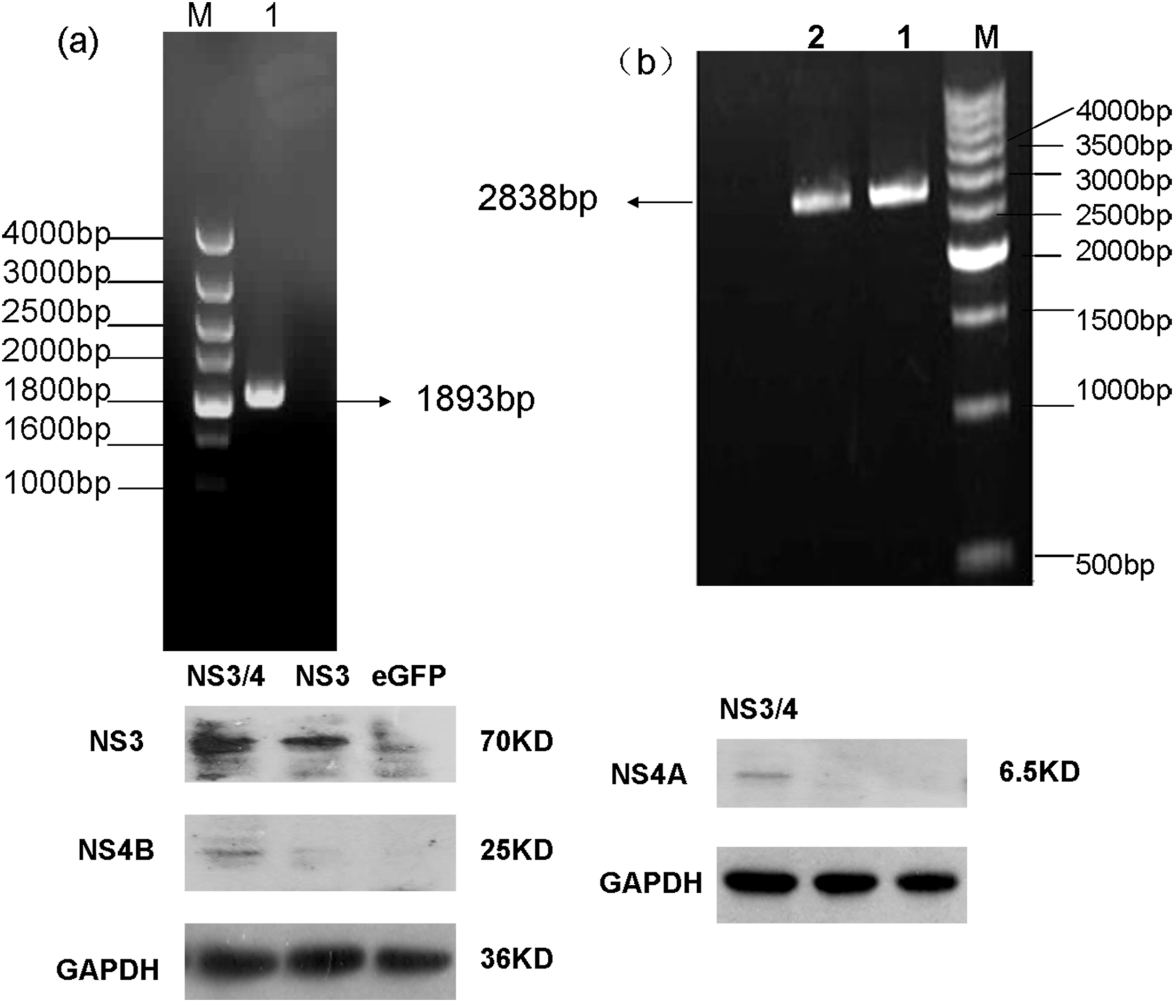
Construction and characterisation of recombinant AAV vectors. (a) Electrophoretic profile of the product of RT-PCR amplification of the NS3 region of HCV. (b) Electrophoretic profile of the product of RT-PCR amplification of the NS3/4 region of HCV. The size of the DNA markers (in bp) is indicated. Arrows indicate 1893-bp and 2838-bp cDNA products. (c) Western blot detection of NS3 and NS3/4 proteins in HepG2 cells 72 h after infection with recombinant AAV. NS3 protein (70 kDa) and NS4B protein (25 kDa) were detected at the expected sizes in a 10% polyacrylamide gel; NS4A protein (6.5 kDa) was detected at the expected size in a 14% polyacrylamide gel. GAPDH (36 kDa) was used as an internal control.

The NS3 and NS3/4 genes were amplified successfully from HCV genotype 1b (Fig 1a and 1b) with specific primers by nested PCR. Recombinant plasmids were constructed by cloning the desired gene into an AAV cis-plasmid. After verification of the recombinant plasmids by restriction enzyme digestion and sequencing, AAV virus was produced by triple plasmid transfection of HEK293 cells and purified by three rounds of caesium chloride gradient centrifugation. The final genome titres of the three AAV vectors were 3.50 × 10^11^ to 1.02 × 10^12^ GC/ml. NS3 and NS3/4 protein expression after AAV virus infection of HepG2 cells was confirmed by western blotting. The bands shown in Fig 1c indicate successful transduction. Additionally, we evaluated the infectivity of AAV by determining the proportion of total cells expressing eGFP by fluorescence microscopy. The infection rate was approxiamtely 60% (data not shown), indicating that AAV is capable of delivering antigens into cells *in vitro* with a relatively high efficiency.

### NS3-specific IgG in sera from AAV-vaccinated mice

**Fig 2:**
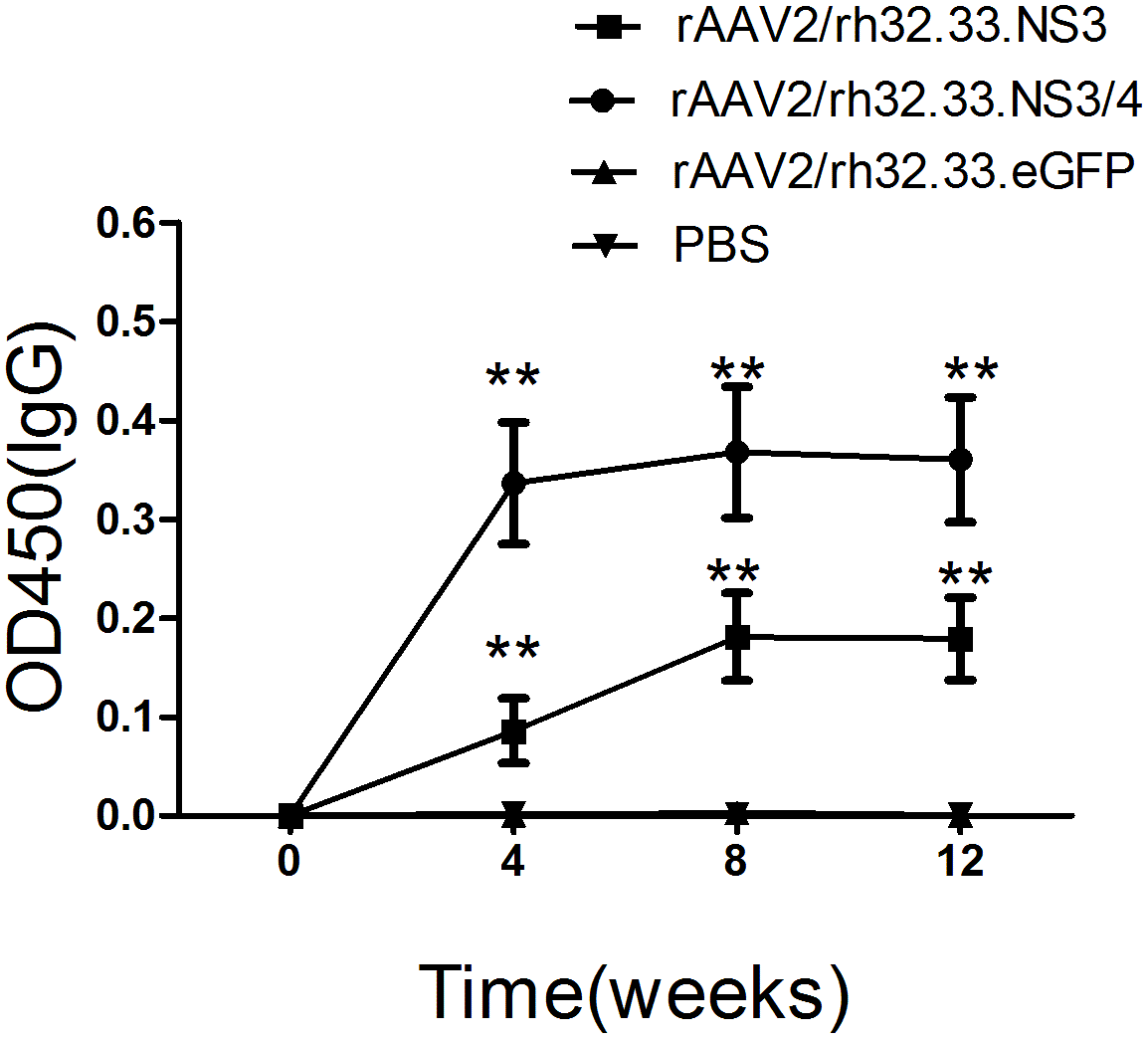
Anti-NS3 IgG antibody levels in sera from AAV-vaccinated mice. Mice were vaccinated with rAAV2/rh32.33.NS3/4, rAAV2/rh32.33.NS3, rAAV2/rh32.33.eGFP, or PBS control. Sera were collected at weeks 0, 4, 8, and 12 and subjected to ELISA. Results are expressed as mean ± SEM optical densities (O.D.) at 450 nm (n = 6). **, p < 0.001.

We assayed NS3-specific IgG in sera from AAV-vaccinated mice by ELISA as an initial assessment of AAV immunogenicity. Four groups of mice were inoculated with equivalent doses of rAAV2/rh32.33.NS3/4, rAAV2/rh32.33.NS3, rAAV2/rh32.33.eGFP, or PBS (control). Blood samples were collected from the retro-orbital sinus at weeks 0, 4, 8, and 12. The AAV vaccines induced a long-lasting antibody response, which was detected up to 12 weeks post-immunisation (Fig 2). At each time point, groups 1 and 2 showed significantly higher NS3-specific IgG antibody levels than did the other two groups (p < 0.001). Importantly, the rAAV2/rh32.33.NS3/4 vaccine induced a rapid antibody response that reached a higher level than that induced by the rAAV2/rh32.33.NS3 vaccine (p = 0.036) (Fig 2).

### IFN-γ, IL-2 and IL-4 mRNAs levels in splenocytes from vaccinated mice

**Fig 3:**
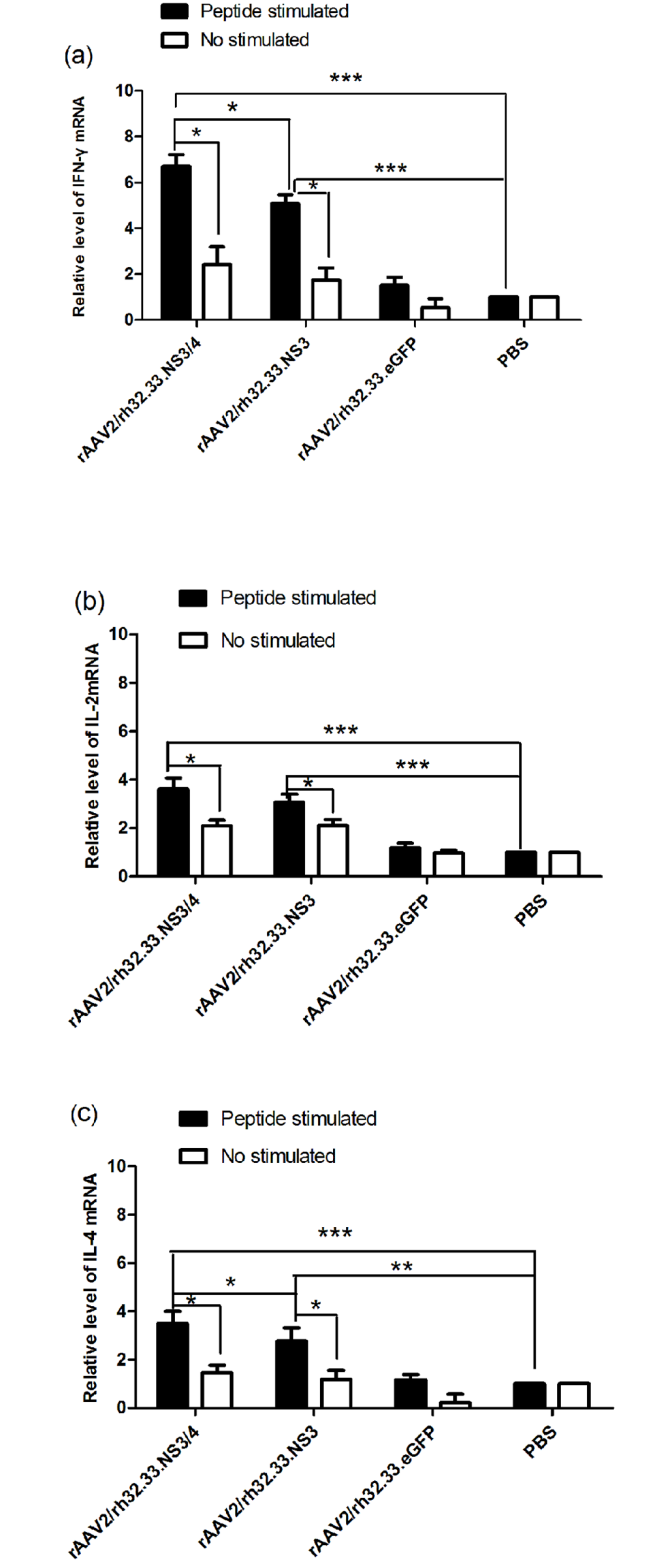
Cytokines mRNA levels in splenocytes from vaccinated mice. (a) IFN-γ, (b) IL-2, and (c) IL-4 mRNA levels in splenocytes from mice were determined at week 4 after vaccination with rAAV2/rh32.33.NS3/4 (group1), rAAV2/rh32.33.NS3 (group2), rAAV2/rh32.33.eGFP (group 3), or PBS (control; group 4). The splenocytes were stimulated with GST-NS3 (peptide stimulated) or PBS (no stimulated) in vitro. IFN-γ, IL-2, and IL-4 mRNA levels were determined by real-time RT-PCR. Data represent mean ± SEM of the data from each group (n = 6 in each group). ***, P<0.0001; *, P<0.05.

To determine whether vaccination with the AAV vectors induced Th1 and Th2 cell-mediated responses *in vivo*, splenocytes were isolated from the mice 4 weeks after immunisation and stimulated with GST-NS3 peptide or PBS for 72 h *in vitro*. The mRNA levels of the Th1 cytokines, (IFN-γ and IL-2) and the Th2 cytokine (IL-4) were then determined by real-time RT-PCR. Results were normalised to the GAPDH mRNA level. IFN-γ, IL-2, and IL-4 mRNA levels were significantly higher in the splenocytes pulsed with GST-NS3 *in vitro* isolated from group 1 and 2 mice vaccinated with rAAV2/rh32.33.NS3/4 or rAAV2/rh32.33.NS3 than in the splenocytes pulsed with the GST-NS3 isolated from the other two groups (Fig 3a–3c). Additionally, the IFN-γ (P = 0.031) and IL-4 (P = 0.016) mRNA levels in the splenocytes pulsed with GST-NS3 *in vitro* isolated from group 1 were significantly higher than those from group 2 (Fig 3a and 3c). However, the IL-2 (P = 0.357) mRNA level was not significantly different between these two groups (Fig 3b). Finally, only in group 1and 2, GST-NS3 stimulation induced significantly higher IFN-γ, IL-2, and IL-4 mRNA levels than PBS control (P<0.05).

### IFN-γ and IL-4 production by splenocytes from vaccinated mice

**Fig 4:**
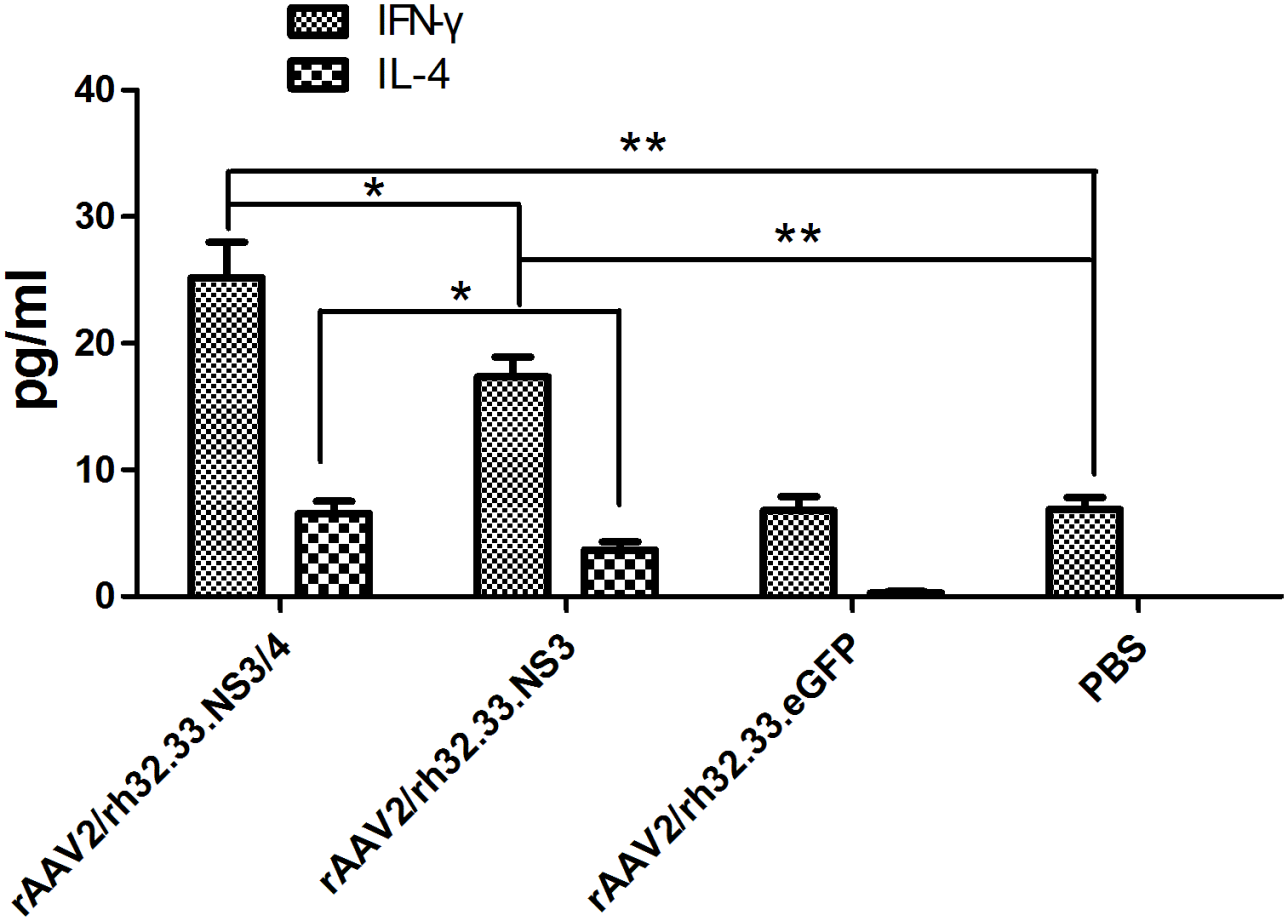
IFN-γ and IL-4 production by splenocytes from vaccinated mice. IFN-γ and IL-4 production by splenocytes of mice were measured at week 4 after vaccination with rAAV2/rh32.33.NS3/4 (group 1), rAAV2/rh32.33.NS3 (group 2), rAAV2/rh32.33.eGFP (group 3), or PBS (group 4). Splenocytes were cultured with NS3 peptide and the supernatants collected for ELISA. Data represent mean ± SEM of the data from each group (n = 6 in each group). ***, P<0.001; *, p <0.05.

To examine the ability of AAV vaccines to induce a cell-mediated response *in vivo*, splenocytes from vaccinated mice were cultured and stimulated with GST-NS3 peptide *in vitro*. Seventy-two hours later, the supernatants were collected and IFN-γ and IL-4 levels measured by ELISA. As seen in Fig 4, splenocytes from group 1 and 2 mice secreted significantly higher IFN-γ and IL-4 levels than did splenocytes from the other groups (p < 0.001). Additionally, splenocytes from group 1 mice secreted higher levels of IFN-γ (p = 0.0356) and IL-4 (p = 0.031) than did those from group 2. Importantly, the IFN-γ level was increased to a significantly greater extent than the IL-4 level in both groups 1 and 2 (P<0.001), which was not indicated by asterisks in Fig 4.

### Analysis of T lymphocyte proliferation from vaccinated mice

**Fig 5:**
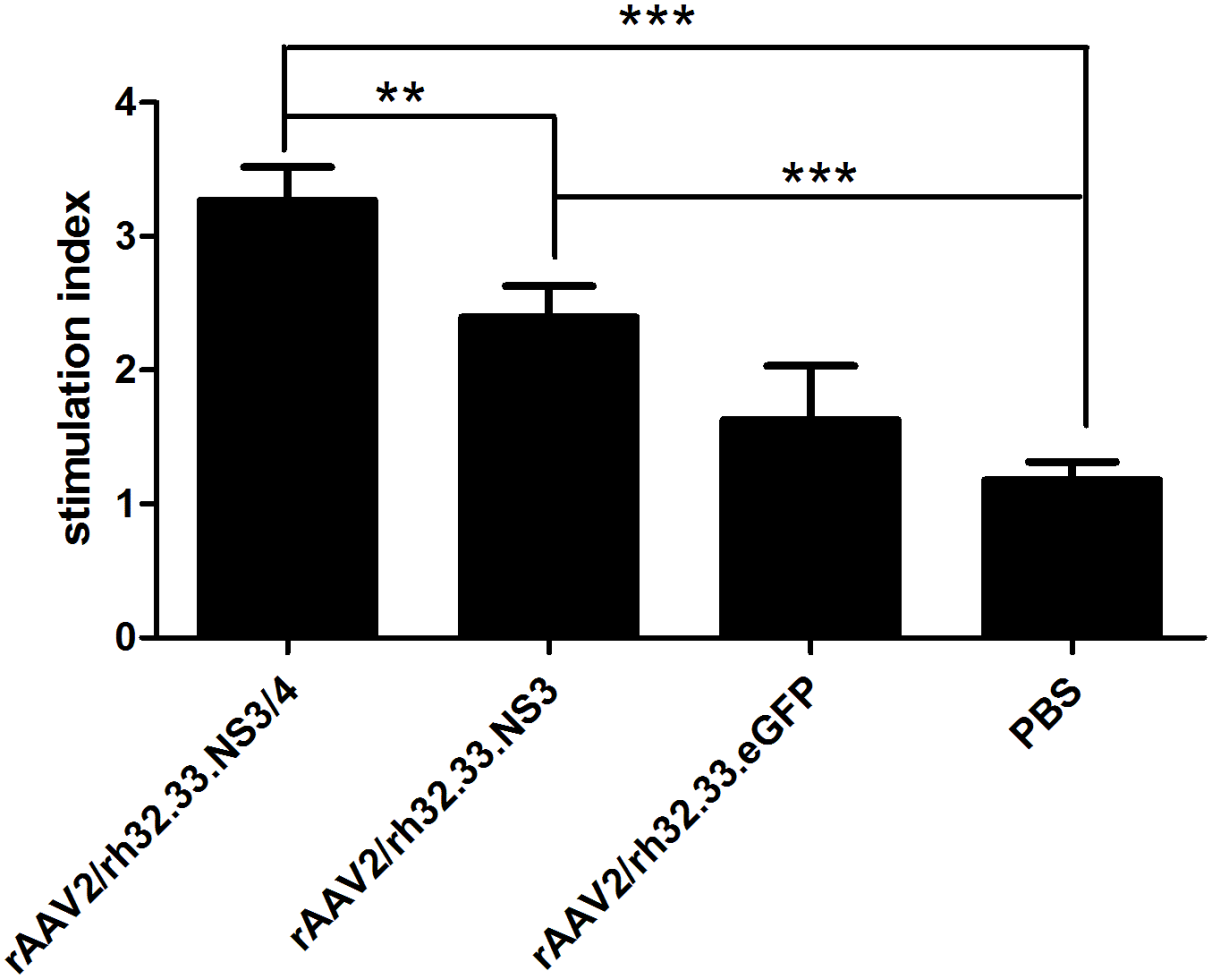
Proliferation of T lymphocytes from vaccinated mice. After vaccination with rAAV2/rh32.33.NS3/4 (group 1), rAAV2/rh32.33.NS3 (group 2), rAAV2/rh32.33.eGFP (group 3), or PBS (control; group 4), splenocytes were cultured with GST-NS3 and then measured by the MTT assay to calculate the SI. Data represent mean ± SEM of the data from each group (n = 6 in each group). ***, P<0.001; **, P<0.01.

Splenic lymphocytes were sorted from the vaccinated mice 4 weeks after immunisation, stimulated with GST-NS3 peptide or PBS for 72 h, and analyzed using MTT assay. As shown in Fig 5, both of rAAV2/rh32.33.NS3/4 vaccine and rAAV2/rh32.33.NS3 vaccine could induce T-cell responses effectively. Notably, these results indicate that rAAV2/rh32.33.NS3/4 vaccine induced stronger T-cell proliferative responses than rAAV2/rh32.33.NS3 vaccine in C57BL/6 mice (P<0.01).

### NS3-specific CTL Activities from vaccinated mice

**Fig 6:**
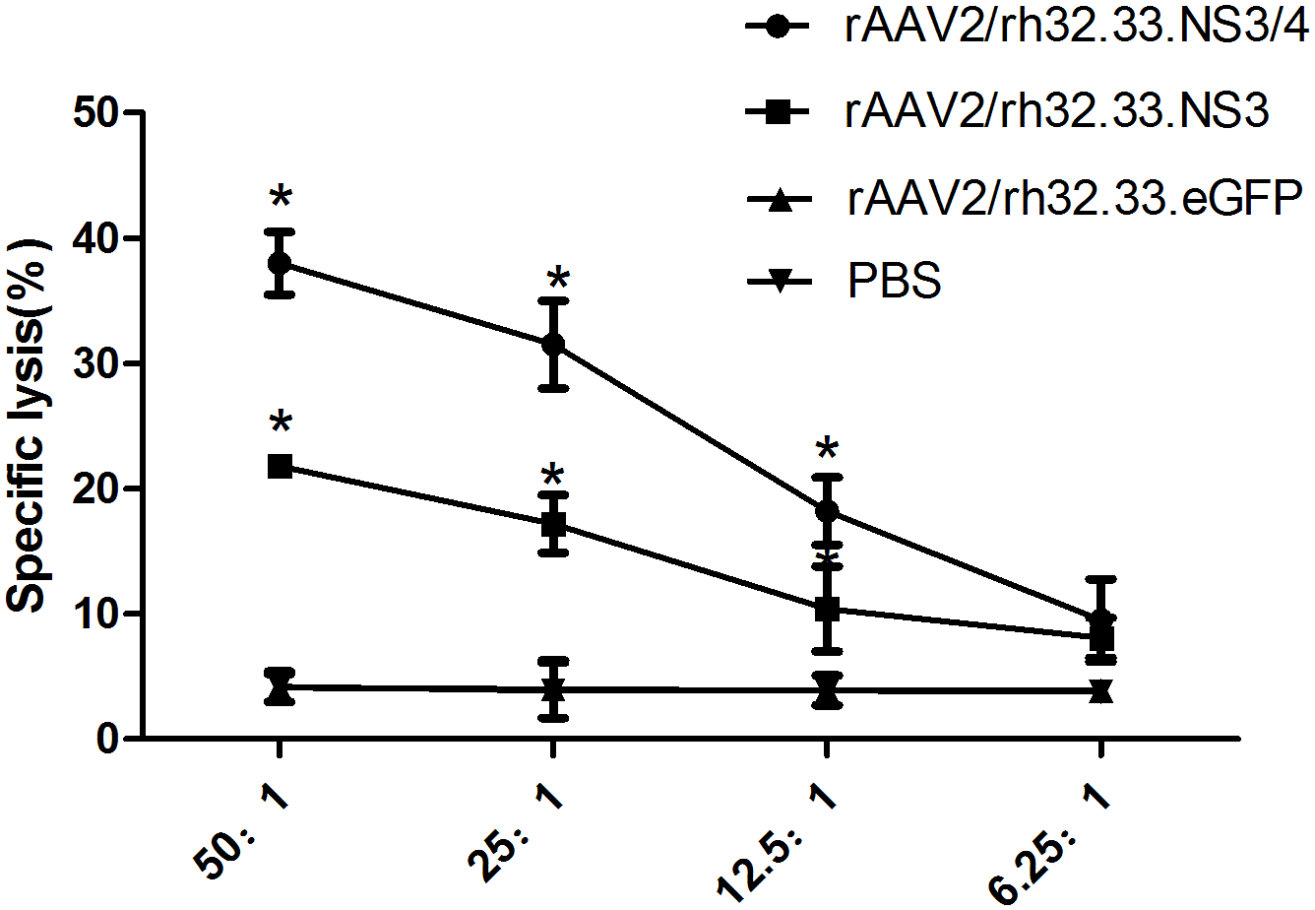
NS3-specific CTL activity in splenocytes from vaccinated mice. Mice were vaccinated with rAAV2/rh32.33.NS3/4 (group 1), rAAV2/rh32.33.NS3 (group 2), rAAV2/rh32.33.eGFP (group 3), or PBS (group 4). Splenocytes were stimulated with GST-NS3 and P815-NS3 cells in vitro. Effectors were cocultured with targets (P815-NS3) for 4h with different ratios of 50:1, 25:1, 12.5:1, and 6.25:1. Released LDH was measured and the percentage of specific killing was calculated. Data represent mean ± SEM of the data from each group (n = 6 in each group). *, P<0.05.

We measured the specific CTL activity induced by each AAV vaccine. Splenocytes obtained from the vaccinated mice were stimulated with GST-NS3 and P815-NS3 cells for 5 days to generate effector cells. Then the effector splenocytes were cocultured with the target P815-NS3 cells to examine the levels of CTL activities. As seen in Fig 6, T lymphocytes from group 1 and 2 mice showed higher specific lysis percentages than did T lymphocytes from the other groups at different E:T ratios except for 6.25:1(P<0.05). Notably, splenocytes from group 1 mice exhibited higher cytolytic activity than did those from group 2 at E:T ratio of 50:1, 25:1, and 12.5:1, respectively (P<0.05).

## Discussion

Despite major progress in the treatment of HCV infection in recent years, development of an effective vaccine remains important. Many candidate HCV vaccines such as synthetic peptides, recombinant proteins, virus-like particles, inactivated yeasts, recombinant viruses and DNA plasmids have been assessed over the past decades [33, 34]. Some are in phase I/II human clinical trials currently, yet no HCV-specific vaccine has reached phase III development [35].

The application of viral vectors to deliver HCV RNA is an appealing option for a vaccine. The majority of viral vector vaccines against HCV tested to date have been based on adenovirus and the modified vaccinia Ankara virus, both of which provide several advantages because of their efficiency of infection of several cell types [36]. It has been shown that MVA (modified vaccinia Ankara virus) boost the HCV-specific T cells induced by ChAd3 optimally and generate high levels of both CD8+ and CD4+ HCV-specific T cells against multiple HCV antigens (NS3, NS4, NS5A, and NS5B) [37]. However, they constitute a safety concern due to the strong inflammatory responses and pre-existing immunity in the human population. Recent studies have indicated that AAV-based vaccines offer potential advantages compared to other virus-based systems because of their intrinsic lack of pathogenicity, ability to infect cells in various tissues, and expression of transgenes at a high and sustained level [38]. Besides, their use for gene therapy has indicated overall absence of toxicity [39]. Based on these benefits, we used the vector of AAVrh32.33 for HCV vaccine, an engineered hybrid between two natural AAV rhesus macaque isolates that possesses a significantly lower seroprevalence and induction of a potent CD8+ T-cell response against the antigen [24, 40]. In this study, AAV vectors encoding two HCV non-structural proteins (AAVrh32.33.NS3 and AAVrh32.33.NS3/4) were constructed. *In vitro*, AAV vectors can infect HepG2 cells with a relatively high efficiency and express HCV NS3 or NS3/4 protein. This is the first report of an AAV vector system capable of expressing HCV antigens.

A vigorous, pathogen-specific adaptive immune response involving both the humoral and cellular branches is required for HCV clearance. HCV-specific CD4^+^ T cells are thought to be essential for generation of an effective immune response, and the reaction to the NS3 and NS4 proteins seems to be vital [18, 19, 41]. Over half of the entire NS3/4 sequence contain CD4^+^ T-cell epitopes that are recognised by certain individuals [15]. The NS3 protein is highly conserved among strains, and numerous T-cell epitopes have been identified within this antigen [42]. Indeed, it has been reported that a positive IFN-γ–secreting response against NS3 protein is related to a reduction in viral load in humans [43]. Therefore, NS3 protein has long been regarded as an ideal antigen for inclusion in an HCV vaccine. The NS4 protein is a required cofactor for the protease activity of NS3. NS4A and NS4B have been found in membrane-associated complexes [44]. The inclusion of NS4A in NS3-based genetic vaccines has been reported to greatly enhance the immunogenicity of NS3 [45]. Additionally, a genotype 1a/1b consensus HCV NS3/NS4A DNA vaccine can reportedly induce strong anti-NS3/NS4A T-cell responses in C57BL/6 mice and rhesus macaques [46]. NS4B is an integral membrane protein localised mainly on the cytoplasmic side of the endoplasmic reticulum [47]. Epitope escape has been demonstrated to contribute to viral persistence [48]. Here, we constructed AAV vectors encoding the full length of the HCV NS3/4 and NS3 proteins and compared their immunogenicity in parallel-vaccinated C57BL/6 mice. We focused on the responses to NS3 peptide stimulation because of the reported significance of NS3-specific T-cell responses in the clearance of acute infection [18, 19]. First, NS3-specific antibody levels were determined to evaluate the humoral immune response. In serum samples from rAAV2/rh32.33.NS3/4-immunised mice, NS3-specific IgG appeared more rapidly and reached higher titres than in rAAV2/rh32.33.NS3-immunised mice (Fig 2), which is an indication of the greater intrinsic immunogenicity of the NS3 protein. Notably, after a single injection, AAV vectors induced sustained antibody levels for up to 12 weeks, suggesting sustained expression of the transgene. Additionally, we found that the level of IL-4, considered as a Th2 cytokine, was significantly higher in splenocytes isolated from the mice vaccinated with rAAV2/rh32.33.NS3/4 than in those isolated from the rAAV2/rh32.33.NS3-vaccinated group (Fig 3c). Adaptive cellular immunity plays a major role in HCV clearance; therefore, whether vaccines induce a cellular immune response to HCV is important in their assessment. In this study, we determined IFN-γ and IL-2 levels as markers of a Th1 response and the IL-4 level as a marker of a Th2 response. As expected, the AAV2/rh32.33.NS3/4 vaccine induced higher IFN-γ and IL-4 levels levels than did the AAV2/rh32.33.NS3 vaccine at both the mRNA and secretion levels (Figs 3a and 4). Moreover, the fact that IFN-γ secretion by splenocytes isolated from AAV-immunised mice was markedly higher than that of IL-4 indicates that AAV vaccines based on HCV NS proteins induce Th1-based cellular immunity, which is required for HCV clearance (Fig 4). The cytolytic CD8+ T lymphocytes are essential effector cells against HCV infection. We have demonstrated that AAV2/rh32.33.NS3/4 vaccine-induced CTL exhibited higher cytolytic activity than did the AAV2/rh32.33.NS3 vaccine, which indicated that the AAV2/rh32.33.NS3/4 vaccine possess good immunogenicity (Fig 6). Overall, our data suggest that an AAV-based vaccine encoding NS3/4 has superior immunogenic properties to an NS3-based vaccine. The possible explanations are as follows. First, NS4 may improve the expression level of NS3 [46]. Second, NS4A prevents NS3 from undergoing rapid degradation in cell culture [49]. Third, NS3/4 may augment more efficient immune epitopes than NS3. However, further experiments are needed to clarify the underlying mechanism.

In conclusion, we developed novel AAV-based anti-HCV genetic vaccines encoding the NS3 and NS3/4 proteins and confirmed their induction of both humoral and cellular immune responses in an experimental mouse model. In the future, we plan to construct AAV-based vaccines encoding other regions of the HCV genome and evaluate their immunogenicity in an available mouse model of HCV infection to develop the most effective possible vaccine. Since their successful application in gene therapy [26], we have reasons to believe that AAV vaccines for HCV are of great value, and future studies will help to define it.

## References

[1] Lauer GM, Walker BD. Hepatitis C virus infection. N Engl J Med. 2001; 345: 41–52. 11439948 10.1056/NEJM200107053450107

[2] Poordad F, McCone J Jr, Bacon BR, Bruno S, Manns MP, Sulkowski MS, et al Boceprevir for untreated chronic HCV genotype 1 infection. N Engl J Med.2011; 364(13):1195–206. 10.1056/NEJMoa1010494 21449783 PMC3766849

[3] Jacobson IM, McHutchison JG, Dusheiko G, Di Bisceglie AM, Reddy KR, Bzowej NH, et al Telaprevir for previously untreated chronic hepatitis C virus infection. N Engl J Med.2011; 364(25):2405–16. 10.1056/NEJMoa1012912 21696307

[4] Afdhal N, Zeuzem S, Kwo P, Chojkier M, Gitlin N, Puoti M, et al Ledipasvir and Sofosbuvir for Untreated HCV Genotype 1 Infection. N Engl J Med.2014; 370(20):1889–98. 10.1056/NEJMoa1402454 24725239

[5] Afdhal N, Reddy KR, Nelson DR, Lawitz E, Gordon SC, Schiff E, et al Ledipasvir and sofosbuvir for previously treated HCV genotype 1 infection. N Engl J Med.2014; 370(16):1483–93. 10.1056/NEJMoa1316366 24725238

[6] Eisenstein M. Vaccines: a moving target. Nature.2011; 474: S16–S17. 10.1038/474S16a 21666730

[7] Habersetzer F, Honnet G, Bain C, Maynard-Muet M, Leroy V, Zarski JP, et al A poxvirus vaccine is safe, induces T-cell responses, and decreases viral load in patients with chronic hepatitis C. Gastroenterology.2011; 141: 890–99. 10.1053/j.gastro.2011.06.009 21699798

[8] Torresi J, Johnson D, Wedemeyer H. Progress in the development of preventive and therapeutic vaccines for hepatitis C virus. J Hepatol. 2011; 54: 1273–85. 10.1016/j.jhep.2010.09.040 21236312

[9] Wedemeyer H, Schuller E, Schlaphoff V, Stauber RE, Wiegand J, Schiefke I, et al Therapeutic vaccine IC41 as late add-on to standard treatment in patients with chronic hepatitis C. Vaccine.2009; 27: 5142–51. 10.1016/j.vaccine.2009.06.027 19559112

[10] Weiland O, Ahlén G, Diepolder H, Jung MC, Levander S, Fons M, et al Therapeutic DNA vaccination using in vivo electroporation followed by standard of care therapy in patients with genotype 1 chronic hepatitis C. Mol Ther.2013; 21:1796–805. 10.1038/mt.2013.119 23752314 PMC3776630

[11] Simmonds P. The origin of hepatitis C virus. Curr Top Microbiol Immunol.2013; 369:1–15. 10.1007/978-3-642-27340-7_1 23463195

[12] Grakoui A, Wychowski C, Lin C, Feinstone SM, Rice CM. Expression and identification of hepatitis C virus polyprotein cleavage products. J Virol.1993; 67: 1385–95. 7679746 10.1128/jvi.67.3.1385-1395.1993 PMC237508

[13] Tang H, Grisé H. Cellular and molecular biology of HCV infection and hepatitis. Clin Sci (Lond) 2009; 117: 49–65. 19515018 10.1042/CS20080631

[14] Reed KE, Rice CM. Overview of hepatitis C virus genome structure, polyprotein processing, and protein properties. Curr Top Microbiol Immunol.2000; 242:55–84. 10592656 10.1007/978-3-642-59605-6_4

[15] Gerlach JT, Ulsenheimer A, Grüner NH, Jung MC, Schraut W, Schirren CA, et al Minimal T-cell-stimulatory sequences and spectrum of HLA restriction of immunodominant CD4+T-cell epitopes within Hepatitis C virus NS3 and NS4 proteins. J Virol. 2005; 79: 12425–33. 16160170 10.1128/JVI.79.19.12425-12433.2005 PMC1211510

[16] Thammanichanond D, Moneer S, Yotnda P, Aitken C, Earnest-Silveira L, Jackson D, et al 2008 Fiber-modified recombinant adenoviral constructs encoding hepatitis C virus proteins induce potent HCV-specific T cell response. Clin Immunol.2008; 128: 329–39. 10.1016/j.clim.2008.04.002 18524682

[17] Diepolder HM, Gerlach JT, Zachoval R, Hoffmann RM, Jung MC, Wierenga EA, et al Immunodominant CD4+ T-cell epitope within nonstructural protein 3 in acute hepatitis C virus infection. J Virol.1997; 71: 6011–19. 9223492 10.1128/jvi.71.8.6011-6019.1997 PMC191858

[18] Diepolder HM, Zachoval R, Hoffmann RM, Wierenga EA, Santantonio T, Jung MC, et al Possible mechanism involving T-lymphocyte response to non-structural protein 3 in viral clearance in acute hepatitisC virus infection. Lancet.1995; 346: 1006–07. 7475549 10.1016/s0140-6736(95)91691-1

[19] Missale G, Bertoni R, Lamonaca V, Valli A, Massari M, Mori C, et al Different clinical behaviors of acute hepatitis C virus infection are associated with different vigor of the anti-viral cell-mediated immune response. J Clin Invest.1996; 98: 706–14. 8698862 10.1172/JCI118842 PMC507480

[20] Cerny A, McHutchison JG, Pasquinelli C, Brown ME, Brothers MA, Grabscheid B, et al Cytotoxic T lymphocyte response to hepatitis C virus-derived peptides containing the HLA A2.1 binding motif. J Clin Invest.1995; 95:521–30. 7860734 10.1172/JCI117694 PMC295505

[21] Casimiro DR, Chen L, Fu TM, Evans RK, Caulfield MJ, Davies ME, et al Comparative immunogenicity in rhesus monkeys of DNA plasmid, recombinant vaccinia virus, and replication-defective adenovirus vectors expressing a human immunodeficiency virus type 1 gag gene. J Virol.2003; 77: 6305–13. 12743287 10.1128/JVI.77.11.6305-6313.2003 PMC154996

[22] Barefoot B, Thornburg NJ, Barouch DH, Yu JS, Sample C, Johnston RE, et al Comparison of multiple vaccine vectors in a single heterologous prime-boost trial. Vaccine.2008; 26: 6108–18. 10.1016/j.vaccine.2008.09.007 18809447 PMC2646904

[23] Draper SJ, Heeney JL. Viruses as vaccine vectors for infectious diseases and cancer. Nat Rev Microbiol.2010; 8:62–73. 10.1038/nrmicro2240 19966816

[24] Lin J, Calcedo R, Vandenberghe LH, Bell P, Somanathan S, Wilson JM. A new genetic vaccine platform based on an adeno-associated virus isolated from a rhesus macaque. J Virol.2009; 83: 12738–50. 10.1128/JVI.01441-09 19812149 PMC2786857

[25] Logan GJ, Wang L, Zheng M, Coppel RL, Alexander IE. Antigen fusion with C3d3 augments or inhibits humoral immunity to AAV genetic vaccines in a transgene-dependent manner. Immunol Cell Biol.2010; 88: 228–32. 10.1038/icb.2009.92 19935770

[26] Mingozzi F, High KA. Therapeutic in vivo gene transfer for genetic disease using AAV: progress and challenges. Nat Rev Genet.2011; 12: 341–55. 10.1038/nrg2988 21499295

[27] Gao GP, Alvira MR, Wang L, Calcedo R, Johnston J, Wilson JM. Novel adeno-associated viruses from rhesus monkeys as vectors for human gene therapy. Proc Natl Acad.2002; 99: 11854–59. 10.1073/pnas.182412299 PMC129358 12192090

[28] Li X, Cao H, Wang Q, Di B, Wang M, Lu J, et al Novel AAV-based genetic vaccines encoding truncated dengue virus envelope proteins elicit humoral immune responses in mice. Microbes Infect.2012; 14: 1000–07. 10.1016/j.micinf.2012.05.002 22626929

[29] Li J, Samulski RJ, Xiao X. Role of highly regulated rep gene expression inadeno-associated virus vector production. J Virol.1997; 71: 5236–43. 9188591 10.1128/jvi.71.7.5236-5243.1997 PMC191759

[30] Xiao X, Li J, Samulski RJ. Efficient long-term gene transfer into muscle tissue of immunocompetent mice by adeno-associated virus vector. J Virol.1996; 70: 8098–108. 8892935 10.1128/jvi.70.11.8098-8108.1996 PMC190884

[31] Livingston BD, Newman M, Crimi C, McKinney D, Chesnut R, Sette A. Optimization of epitope processing enhances immunogenicity of multiepitope DNA vaccines. Vaccine.2001; 19, 4652–60. 11535313 10.1016/s0264-410x(01)00233-x

[32] Fournillier A, Dupeyrot P, Martin P, Parroche P, Pajot A, Chatel L, et al Primary and memory T cell responses induced by hepatitis C virus multiepitope long peptides. Vaccine.2006; 24, 3153–64. 16481078 10.1016/j.vaccine.2006.01.039

[33] Roohvand F, Kossari N. Advances in hepatitis C virus vaccines, part two: advances in hepatitis C virus vaccine formulations and modalities. Expert Opin Ther Pat.2012; 22:391–415. 10.1517/13543776.2012.673589 22455502

[34] Liang TJ. Current progress in development of hepatitis C virus vaccines. Nat Med 2013, 19:869–78. 10.1038/nm.3183 23836237 PMC6263146

[35] Law LMJ, Landi A, Magee WC, Lorne Tyrrell D, Houghton M. Progress towards a hepatitis C virus vaccine. Emerging Microbes & Infections.2013; 2:e79. 26038445 10.1038/emi.2013.79 PMC3924556

[36] Fournillier A, Gerossier E, Evlashev A, Schmitt D, Simon B, Chatel L, et al An accelerated vaccine schedule with a poly-antigenic hepatitis C virus MVA-based candidate vaccine induces potent, long lasting and in vivo cross-reactive T cell responses. Vaccine.2007; 25: 7339–53. 17875349 10.1016/j.vaccine.2007.08.020

[37] Swadling L, Capone S, Antrobus RD, Brown A, Richardson R, Newell EW, et al A human vaccine strategy based on chimpanzee adenoviral and MVA vectors that primes, boosts, and sustains functional HCV-specific T cell memory. Sci Transl Med.2014; 6: 261ra 153. 10.1126/scitranslmed.3009185 PMC4669853 25378645

[38] Nieto Karen, Salvetti Anna. AAV vectors vaccines against infectious diseases. Front Immunol.2014; 5: 5 10.3389/fimmu.2014.00005 24478774 PMC3896988

[39] Dismuke DJ, Tenenbaum L, Samulski RJ. Biosafety of recombinant adeno-associated virus vectors. CurrGeneTher.2013 13:434–52. 10.2174/15665232113136660007 24195602

[40] Vandenberghe LH, Breous E, Nam HJ, Gao G, Xiao R, Sandhu A, et al Naturally occurring singleton residues in AAV capsid impact vector performance and illustrate structural constraints. Gene Ther.2009; 16: 1416–28. 10.1038/gt.2009.101 19727141 PMC2795093

[41] Rehermann B. Hepatitis C virus versus innate and adaptive immune responses: a tale of coevolution and coexistence. J Clin Invest.2009; 119:1745–54. 10.1172/JCI39133 19587449 PMC2701885

[42] Habersetzer F, Baumert TF, Stoll-Keller F. GI-5005, a yeast vector vaccine expressing an NS3-core fusion protein for chronic HCV infection. Curr Opin Mol Ther.2009; 11: 456–62. 19649991

[43] Habersetzer F, Honnet G, Bain C, Maynard-Muet M, Leroy V, Zarski JP, et al A Poxvirus vaccine is safe, induces T-cell responses,and decreases viral load in patients with chronic Hepatitis C. Gastroenterology.2011; 141: 890–99. 10.1053/j.gastro.2011.06.009 21699798

[44] Huang Y, Uchiyama Y, Fujimura T, Kanamori H, Doi T, Takamizawa A, et al A human hepatoma cell line expressing hepatitis c virus nonstructural proteins tightly regulated by tetracycline. Biochem Biophys Res Commun.2001; 281: 732–40. 11237719 10.1006/bbrc.2001.4424

[45] Frelin L, Alheim M, Chen A, Söderholm J, Rozell B, Barnfield C, et al Low dose and gene gun immunization with a hepatitis C virus nonstructural (NS) 3 DNA-based vaccine containing NS4A inhibit NS3/4A-expressing tumors in vivo. Gene Ther.2003; 10: 686–99. 12692597 10.1038/sj.gt.3301933

[46] Lang KA, Yan J, Draghia-Akli R, Khan A, Weiner DB. Strong HCV NS3- and NS4A-specific cellular immune responses induced in mice and Rhesus macaques by a novel HCV genotype 1a/1b consensus DNA vaccine. Vaccine.2008; 26:6225–31. 10.1016/j.vaccine.2008.07.052 18692108 PMC4477808

[47] Hügle T, Fehrmann F, Bieck E, Kohara M, Kräusslich HG, Rice CM, et al The hepatitis C virus nonstructural protein 4B is an integral endoplasmic reticulum membrane protein. Virol.2001; 284: 70–81. 10.1006/viro.2001.0873 11352669

[48] Timm J, Lauer GM, Kavanagh DG, Sheridan I, Kim AY, Lucas M, et al CD8 epitope escape and reversion in acute HCV infection. J Exp Med.2004; 200:1593–604. 15611288 10.1084/jem.20041006 PMC2212005

[49] Tanji Y, Hijikata M, Satoh S, Kaneko T, Shimotohno K. Hepatitis C virus-encoded nonstructural protein NS4A has versatile functions in viral protein processing. J Virol.1995; 69: 1575–81. 7853491 10.1128/jvi.69.3.1575-1581.1995 PMC188752

